# Non-Thermal Supercritical Carbon Dioxide Processing Retains the Quality Parameters and Improves the Kinetic Stability of an Araticum Beverage Enriched with Inulin-Type Dietary Fibers

**DOI:** 10.3390/foods12132595

**Published:** 2023-07-04

**Authors:** Henrique Silvano Arruda, Eric Keven Silva, Glaucia Maria Pastore, Mario Roberto Marostica Junior

**Affiliations:** 1 Department of Food Science and Nutrition, School of Food Engineering, University of Campinas, Monteiro Lobato Street 80, Campinas 13083-862, SP, Brazil; glaupast@unicamp.br (G.M.P.); mmarosti@unicamp.br (M.R.M.J.); 2 Department of Food Engineering and Technology, School of Food Engineering, University of Campinas, Monteiro Lobato Street 80, Campinas 13083-862, SP, Brazil; ekeven@unicamp.br

**Keywords:** emerging technology, green chemistry, food stabilization, fructooligosaccharides, functional food, prebiotic, *Annona crassiflora* Mart., marolo, Brazilian biodiversity, Cerrado fruit

## Abstract

Fruit-based beverages have been considered excellent food vehicles for delivering prebiotics. However, the conventional thermal processes currently used to microbiologically and enzymatically stabilize these products may cause significant losses in their sensory, physicochemical, nutritional, and bioactive characteristics. Thus, in this study, we evaluate the effect of different levels of pressure (8, 15, and 21 MPa) and temperature (35 and 55 °C) on the characteristics of an inulin-enriched araticum beverage processed with non-thermal supercritical carbon dioxide (SC–CO_2_) technology. The temperature showed a significant effect on total soluble solids, pH, particle size distribution, and kinetic stability. In contrast, pressure affected only the particle size distribution. The interaction between pressure and temperature influenced the total soluble solids, pH, and particle size distribution. Color parameters, ζ-potential, and glucose and fructose contents were not modified after all SC–CO_2_ treatments. Moreover, the SC–CO_2_ treatments preserved the inulin molecular structure, thus maintaining its prebiotic functionality. Overall, the SC–CO_2_ treatment did not alter the sensory, nutritional, and functional quality of the beverage, while improving its physical stability during storage. Therefore, non-thermal SC–CO_2_ treatment can be an alternative to current conventional processes for stabilizing inulin-enriched fruit-based beverages.

## 1. Introduction

Brazil is home to one of the world’s largest biodiversities, accounting for approximately 15 to 20% of the global biological diversity, and it is at the top among 17 megadiverse countries in the world [1]. Furthermore, it contains two biodiversity hotspots (the Atlantic Forest and the Cerrado biomes) and is the second country in terms of the richness of endemic species, second only to Indonesia [2]. However, only approximately 11% of Brazilian biodiversity has been cataloged [3], and many native fruit species remain unknown and/or unexplored [4]. These peculiarities offer a wide range of opportunities in the search for plants/fruits with sensory, nutritional, and functional appeal.

Among the native Brazilian fruits that have a high potential for economic and technological exploration, but remain underutilized or even unknown, we can highlight araticum (*Annona crassiflora* Mart.). Araticum is a native and endemic fruit of the Brazilian Cerrado, belonging to the Annonaceae family. This species is among the 20 most commonly used foods in regional cuisine and it has been used for centuries in folk medicine for the treatment of several pathological conditions (e.g., pain, rheumatism, diarrhea, tumors, Chagas disease, snake bites, and skin and scalp infections, among others) [5]. Furthermore, its fruits have attractive sensory characteristics (appealing color, intense flavor, and exotic aroma), as well as significant nutritional potential (providing a good source of dietary fibers, sugars, vitamins A and C, folates, and minerals such as copper, manganese, potassium, and zinc) and functional properties (high content of antioxidants, mainly phenolic compounds and carotenoids) [6]. Recent studies have demonstrated that the edible part of this fruit exhibits antioxidant [7,8,9], anti-inflammatory [10], anticancer [11], anti-Alzheimer’s [12], and antibacterial activities [13,14], which may be directly related to the presence of different bioactive compounds found in araticum pulp, particularly phenolic compounds, alkaloids, annonaceous acetogenins, and carotenoids [5]. Nonetheless, araticum is a seasonal and highly perishable fruit, which hinders its availability throughout the year and the preservation of its postharvest freshness, making its consumption possible only during certain times of the year and in limited regions [5,6]. Therefore, one way to increase the availability of and add more value to this fruit is by processing its pulp and/or developing new food products, such as fruit-based beverages.

In addition to being a good source of vitamins, antioxidants, bioactive compounds, and minerals, fruit-based beverages are considered excellent vehicles for prebiotics. They are refreshing, have hydration properties, and present attractive sensory characteristics, making them readily accepted and frequently consumed by a significant proportion of the population [15]. Currently, a prebiotic has been defined as “a substrate that is selectively utilized by host microorganisms, conferring a health benefit” [16]. Several non-digestible carbohydrates have been reported to exert a prebiotic effect, but fructan-type (inulin and fructooligosaccharides) and galactan-type oligosaccharides are the most extensively documented dietary prebiotics that provides health benefits to humans [17].

Inulin is one of the most extensively studied and commercially available non-digestible carbohydrates with recognized prebiotic claims [18]. This carbohydrate has been successfully added to different beverages to improve their technological and functional properties [19,20]. Inulin is composed of fructan-type chains with different degrees of polymerization (typically ranging from 2 to 60). It consists primarily, if not exclusively, of repeated units of fructose linked together by β-(2→1) bonds, usually with a typical terminating glucose molecule [21]. The prebiotic claims of inulin are due to its characteristic chemical structure. The β-(2→1)-glycosidic bonds formed between the successive fructose units that compose inulin are not hydrolyzed by human digestive enzymes, allowing this carbohydrate to reach the colon. Here, it is degraded by β-fructanase enzymes that are prevalent in health-promoting bacteria (primarily, but not exclusively, *Bifidobacterium* and *Lactobacillus*), and metabolized by these microorganisms, providing various beneficial effects in terms of the host’s health and well-being [18]. Therefore, the prebiotic effects of inulin-enriched food products depend on the preservation of the native chemical structure of this non-digestible carbohydrate.

In this sense, the development of a new beverage based on the addition of inulin to fruit-based beverages as a liquid carrier medium would be a promising health-promoting food product. However, the conventional thermal treatments currently used to stabilize fruit-based beverages can promote losses in their nutritional and bioactive properties due to the degradation of thermosensitive compounds (e.g., vitamins, ascorbic acid, phenolic compounds, antioxidants, proteins, and carbohydrates, among others). This can reduce their sensory quality through the formation of off-flavors, a “cooked taste”, and non-enzymatic browning [18,22]. Thus, one of the greatest challenges facing the food industry today is the development of technologies that ensure the safety of food products while preserving their sensory, nutritional, and functional attributes, similar to those of unprocessed products [23]. In this context, SC–CO_2_ technology has emerged as an alternative to conventional thermal processing methods applied to stabilize food and beverages, as this technology is capable of efficiently and non-thermally inactivating spoilage microorganisms and food-deteriorating enzymes [24,25]. Furthermore, recent studies have demonstrated the technical and economic feasibility of implementing SC–CO_2_ technology on an industrial scale [26]. Hence, non-thermal SC–CO_2_ technology can be a solution for the stabilization of functional beverages with thermosensitive components. Although SC–CO_2_ processing is recognized as an efficient technology for inactivating unwanted enzymes and microorganisms in foods, the effects of this technology on other characteristics of food products, particularly fruit-based beverages enriched with prebiotic carbohydrates, have been poorly studied. Likewise, there is scarce literature discussing the impacts of high-pressure carbon dioxide on the kinetic stability of juices and beverages. Therefore, this study aimed to evaluate the impact of non-thermal processing by using SC–CO_2_ technology on the quality parameters of a functional inulin-enriched araticum beverage. We studied the effects of pressure (8, 15, and 21 MPa) and temperature (35 and 55 °C) on some physicochemical properties (total soluble solids, pH, and ζ-potential), color parameters, particle size distribution, kinetic stability, content of sugars, and inulin structure preservation of the functional araticum beverage.

## 2. Materials and Methods

### 2.1. Chemicals and Reagents

The standards of glucose, fructose, and sucrose were obtained from Sigma-Aldrich Chemical Co.^®^ (St. Louis, MO, USA), whereas the standards of fructooligosaccharides (FOS) including 1-kestose (GF_2_), 1-nystose (GF_3_), and 1-fructofuranosylnystose (GF_4_) were provided by Wako Pure Chemical Industries^®^ (Osaka, Japan). Sodium acetate and sodium hydroxide solution (50%) for ionic chromatography were purchased from Sigma-Aldrich Chemical Co.^®^ (St. Louis, MO, USA). The water used was obtained from a Milli-Q water purification system (Millipore^®^, Bedford, MA, USA). All other solvents and reagents used in this study were of analytical grade.

### 2.2. Plant Material and Sample Preparation

The completely mature araticum fruits were collected in natural areas of the Cerrado biome, located in the city of Carmo do Paranaíba (19°00′03″ south latitude, 46°18′58″ west longitude, and 1061 m altitude), Minas Gerais, Brazil. The fruits were washed and manually peeled and pulped. Then, the pulp was freeze-dried (LIOTOP^®^, model L101, São Carlos, Brazil), ground using a knife grinder (Marconi^®^, model MA340, Piracicaba, Brazil), and stored at −20 °C until analysis.

A voucher specimen (UEC 197249) was deposited in the Herbarium of the Institute of Biology of the University of Campinas, Brazil (Herbarium UEC). The Genetic Heritage Management Board (CGen) under number A437549, following Law n° 13.123/2015 and its regulations, regimented the activity of access to Genetic Heritage.

### 2.3. Functional Araticum Beverage Formulation

The functional araticum beverage was produced with freeze-dried araticum pulp (7 g/100 g), inulin GR (3 g/100 g, BENEO-Orafti^®^, São Paulo, Brazil) with a mean degree of polymerization (DP) = 10, and ultrapure water (90 g/100 g, Millipore^®^, Bedford, MA, USA). The inulin amount added to the beverage was established so that the araticum beverage had prebiotic claims (3−8 g inulin per serving, considering a serving of 200 mL beverage/day) [18]. For beverage manufacturing, initially, araticum pulp powder was reconstituted in approximately half of the volume of water at room temperature. The inulin was dissolved in the remaining volume of hot water (80 °C), then rapidly cooled until 40 °C was reached, using a cold bath. It was then incorporated into the reconstituted araticum pulp. The mixture was homogenized with the aid of a blender (MX1500 Waring^®^, Stamford, CT, USA). The functional araticum beverage presented total soluble solids of 7.4 ± 0.1 °Brix. The beverage samples used as the control were named “untreated”.

### 2.4. Non-Thermal SC–CO_2_ Processing

The functional araticum beverage was processed using an SC–CO_2_ unit that was previously described and validated by Silva et al. [27]. There was a modification in the system output in the present study, in which the reactor was firstly depressurized, and the beverage was taken out after the total depressurization of the system. SC–CO_2_ processing was performed by loading 315 mL of the functional araticum beverage into a 630 mL stainless-steel reactor (67.85 mm inner diameter and 240 mm height) coupled with a digital thermometer. CO_2_ with a purity of ≥99.9% (Gama Gases Especiais Ltda.^®^, São Bernardo do Campo, Brazil), cooled at −6 °C using a thermostatic bath (Marconi^®^, model MA-184, Piracicaba, Brazil) and pressurized by using a pneumatic pump (Maximator^®^, model M-111 L, Nordhausen, Germany), was pumped to the high-pressure reactor, promoting the homogenization of the sample until the operating pressure was reached. All experiments were carried out with a CO_2_ volume ratio of 50% and a processing time of 10 min, according to previous works [22,27,28]. The processing time was counted only when the system reached the operating temperature and pressure according to each experimental condition studied. At the end of each treatment, the reactor was depressurized, and the functional araticum beverage was collected from the reactor and immediately cooled (<30 °C).

The pressure levels were selected based on the operating limit of the assembled SC–CO_2_ unit (8−21 MPa). Meanwhile, the temperatures were set with the aim of evaluating supercritical stabilization as a non-thermal process (<60 °C). Thus, the effects of pressure (8, 15, and 21 MPa) and temperature (35 and 55 °C) on the pH, total soluble solids, ζ-potential, particle size distribution, color parameters, kinetic stability, glucose content, fructose content, short-chain FOS content, and inulin profile were investigated using a full factorial experimental design (3 × 2). All experiments were performed in duplicate, accounting for 12 runs.

### 2.5. pH and Total Soluble Solids (TSS) Analysis

The pH was determined using a digital potentiometer (Digmed^®^, model DM-22, Digicrom Analytical, São Paulo, Brazil). TSS was measured with a digital refractometer (Atago Brasil^®^, model PAL-1, Ribeirão Preto, Brazil). The analyses were carried out in duplicate at 25 ± 1 °C.

### 2.6. ζ-Potential Measurement

The surface charges of the functional araticum beverage were determined by measuring the ζ-potential using a chamber of microelectrophoresis (ZetaSizer Nano-ZS, Malvern Instruments Ltd.^®^, Worcestershire, UK). The beverage samples were diluted to 1:100 in deionized water before the analysis. The measurements were performed in triplicate at 25 ± 1 °C.

### 2.7. Particle Size Distribution

The particle size distribution of the functional araticum beverage was characterized using a laser light diffraction instrument, the Mastersizer 2000 (Malvern Instruments Ltd.^®^, Malvern, UK). The beverages were dispersed in distilled water and the measurements were realized in triplicated right after their preparation or SC–CO_2_ processing. The mean particle size was expressed as the volume-based mean diameter (D_4,3_) according to Equation (1) [28].

(1) $$D_{4,3}=\frac{{\Sigma n}_{i}d_{i}^{4}}{{\Sigma n}_{i}d_{i}^{3}}$$

where n_i_ is the number of particles with a diameter d_i_.

### 2.8. Kinetic Stability

The physical stability of the beverages was evaluated by the phase separation kinetics by using the technique of near-infrared light backscattering at 880 nm (Turbiscan Lab^®^ Expert, Formulation, Toulouse, France). Immediately after the processing, aliquots (20 mL) of each treatment were transferred to flat-bottom cylindrical glass tubes (16 mm diameter and 40 mm height) for analysis of the backscattering profile. The tubes were sealed and stored under refrigerated conditions (5 ± 2 °C) and the backscattering profiles were measured immediately after the SC–CO_2_ processing (day 0), and after 1 (day 1), 3 (day 3), and 7 days (day 7). The global Turbiscan Stability Index (TSI) was calculated by using Turbisoft software version 2.0.0.28 (Formulaction^®^, Toulouse, Haute-Garonne, France).

### 2.9. Color Parameters

The effect of the SC–CO_2_ processing on the functional araticum beverage color was evaluated using a colorimeter (Konica Minolta Camera Co. Ltd.^®^, model CR-400, Osaka, Japan). The color analysis was carried out based on the L* (brightness/darkness), C* (Chroma), h (hue angle), a* (redness/greenness), and b* (yellowness/blueness), according to the CIE (Commission Internationale de l’Eclairage). The color index (CI), yellow index (YI), and browning index (BI) were calculated according to Equations (2)−(5) [29,30].

(2) $$\mathrm{CI}=\frac{180-h}{L^{*}-C^{*}}$$

(3) $$\mathrm{YI}=\frac{142.86b^{*}}{L^{*}}$$

(4) $$\mathrm{BI}=\frac{100\times \left(x-0.31\right)}{0.172}$$

(5) $$x=\frac{a^{*}+1.75L^{*}}{5.645L^{*}+a^{*}-3.012b^{*}}$$

To better visualize the difference between the SC–CO_2_ treatments, the color difference (ΔE*) was calculated according to Equation (6) [28].

(6) $${\Delta E}^{*}=\sqrt{{\left({\Delta L}^{*}\right)}^{2}+{\left({\Delta C}^{*}\right)}^{2}+{\left(\Delta h\right)}^{2}}$$

where Δ represents the parameter difference between the SC–CO_2_-processed and untreated beverage samples.

### 2.10. Determination of Sugars and FOS by HPAEC–PAD

The profile and content of sugars and FOS in the functional araticum beverage were determined by high-performance anion exchange chromatography coupled with pulsed amperometric detection (HPAEC–PAD) using an ion chromatographer Dionex ICS-5000 (Thermo Fisher Scientific^®^, Waltham, MA, USA), according to the method described by Silva et al. [21].

### 2.11. Statistical Analysis

The effects of SC–CO_2_ processing on the physicochemical properties, kinetic stability, and content of sugars of the functional araticum beverage according to the proposed experimental design were analyzed by using Minitab software version 18.0 (Minitab Inc.^®^, State College, PA, USA). Analyses of variance (ANOVA) were performed at a significance level of 5% (*p*-value < 0.05).

## 3. Results and Discussion

The maintenance of the physical and chemical characteristics of post-processing food products is essential for retaining their functional properties and sensory acceptance by consumers. Processes that lead to significant changes in these characteristics can harm the sensory acceptance of food products and their potential biological activities. Here, we evaluated the synergistic impact between different combinations of pressure and temperature on the physicochemical (pH, TSS, and ζ-potential), physical (particle size distribution and color parameters), and chemical (sugars/FOS profile and content) characteristics of a functional araticum beverage processed with SC–CO_2_ technology. The effects of these variables on the beverage will be discussed in detail in the following sections.

### 3.1. pH

The pH is a physicochemical parameter that should be monitored in processed beverages, as changes in this post-processing parameter can impact the physical stability of the beverage, the chemical stability of compounds (particularly bioactive compounds), and the sensory acceptance of the beverage. As is seen in Table 1, the functional araticum beverages showed similar pH values (4.43–4.54). However, the interaction between pressure and temperature significantly affected the beverage’s pH values (*p*-value = 0.045). When the temperature was fixed at 35 °C, the pH increased progressively (4.43–4.52) as the pressure was elevated. On the other hand, the pH decreased progressively (4.54–4.5) as the pressure was elevated, when the processing temperature was 55 °C. Ramírez-Rodrigues et al. [31] also observed a significant change in the pH of an SC–CO_2_-treated hibiscus beverage (34.5 MPa, 8% CO_2_, 6.5 min, and 40 °C). Despite a significant effect of the interaction between pressure and temperature on the beverage’s pH, this effect was only slightly significant (*p*-value = 0.045, which is very close to 0.05), and therefore the effect of the interaction of these variables on the beverage’s pH can be neglected. Moreover, the final pH of the beverage subjected to different SC–CO_2_ treatments (4.43–4.54) was very close to the pH of the untreated beverage (4.5), corroborating most studies on beverages processed with SC–CO_2_, which have reported no modification of the beverage’s pH post-SC–CO_2_ processing [21,22,28,32,33]. The slight but significant change in the pH values of the functional araticum beverage after the SC–CO_2_ treatments is possibly due to the presence of a low amount of residual CO_2_ in the beverage after system depressurization. This residual CO_2_ tends to form carbonic acid (H_2_CO_3_) in an aqueous media, which then dissociates into bicarbonate (HCO_3_^−^), carbonate (CO_3_^2−^), and hydrogen (H^+^) ions, altering the beverage’s pH [21,33].

### 3.2. Total Soluble Solids (TSS)

TSS is an essential indicator of sugar content in plant-based beverages. Therefore, changes in TSS values can indicate some type of structural modification of carbohydrates present in a beverage, such as hydrolysis of polysaccharides or degradation of sugars. Moreover, significant changes in the TSS can affect the sensory preference for beverages. As is shown in Table 1, the TSS of the SC–CO_2_-treated functional araticum beverage was significantly influenced by both temperature (*p*-value = 0.041), and the interaction between pressure and temperature (*p*-value = 0.041). The TSS of the beverage increased progressively as the temperature increased. On the other hand, when the processing temperature was fixed at 35 °C, the TSS of the beverage decreased as the pressure was increased from 8 to 15 MPa (from 7.4 to 7.1 °Brix), but increased again when the pressure was raised to 21 MPa (from 7.1 to 7.3 °Brix). The opposite behavior was observed for the temperature of 55 °C, where the TSS increased as the pressure was raised from 8 to 15 MPa (from 7.4 to 7.5 °Brix), but decreased again when the pressure was raised to 21 MPa (from 7.5 to 7.4 °Brix). Once again, as was discussed earlier for the pH response variable, although the statistics indicated a significant effect of temperature, and the interaction between pressure and temperature on the TSS of the SC–CO_2_-treated functional araticum beverage, these effects were only marginally significant (*p*-value = 0.041, which is very close to 0.05) and, therefore, they can be neglected. Moreover, the final TSS of the beverages subjected to the different SC–CO_2_ treatments (7.1−7.5 °Brix) was very close to the TSS of the untreated beverage (7.4 °Brix), supporting this hypothesis. In fact, most studies conducted using SC–CO_2_ technology in beverage processing have indicated the absence of an impact of these variables on TSS [21,22,28,32]. This result suggests that SC–CO_2_ treatment maintains the chemical structure of the carbohydrates present in the post-processing functional araticum beverage. The effect of SC–CO_2_ treatments on the qualitative and quantitative profile of sugars present in the beverage will be discussed in more detail in Section 3.7.

The slight but significant increase in the TSS of the SC–CO_2_-treated beverage with the increase in temperature may be due to the increased solubilization of some solutes, deflocculation of macromolecules, and/or hydrolysis/release of components from the plant material cell wall (e.g., lignocellulosic matter) caused by the temperature increase [34]. When the functional araticum beverage was subjected to SC–CO_2_ treatment with the low-temperature level (35 °C), a reduction in TSS was initially observed by increasing the pressure from 8 to 15 MPa (from 7.4 to 7.1 °Brix). This was followed by an increase when the pressure was further increased from 15 to 21 MPa (from 7.1 to 7.3 °Brix). During SC–CO_2_ treatment, a temporary reduction in the beverage’s pH occurs inside the high-pressure reactor due to the solubilization of CO_2_ in the beverage, which leads to the formation of carbonic acid (H_2_CO_3_). In turn, this dissociates in the aqueous medium, releasing hydrogen ions (H^+^). However, after processing, CO_2_ is removed from the beverage by depressurizing the system, and the pH returns to its initial value. [22]. Initially, the increase in processing pressure associated with the temporary reduction in the beverage’s pH during SC–CO_2_ treatment may promote the precipitation of charged proteins and polymers. This may lead to the formation of aggregates and, consequently, to the dragging of soluble solids that become undetectable [28]. However, the progressive increase in pressure leads to an elevation in the system tension during SC–CO_2_ processing. In turn, the increased tension of the system causes the breakdown of both protein/polymer aggregates and cell wall fragments into smaller units, solubilizing them in the system and promoting an increase in the TSS of the beverage [21]. On the other hand, the highest processing temperature level (55 °C) potentiates the effect of pressure on the breakdown of protein/polymeric aggregates and cell wall fragments, initially increasing the TSS of the beverage as the system pressure is increased from 8 to 15 MPa (from 7.4 to 7.5 °Brix). However, the intensification of SC–CO_2_ treatment (association between higher temperature (55 °C) and pressure (21 MPa) levels) can lead to the degradation of sugars through chemical reactions such as the Maillard, caramelization, and oxidation reactions, promoting a reduction in the TSS of the beverage (from 7.5 to 7.4 °Brix) [18,21].

### 3.3. ζ-Potential

The ζ-potential describes the magnitude of the surface charge density of the molecules present in a system. Changes in the ζ-potential can indicate some type of structural modification of the compounds present in a beverage and thus affect its physical stability, shelf life, and bioactivity. Table 1 shows the mean values of the ζ-potential of the untreated and SC–CO_2_-treated functional araticum beverages. According to the statistical analysis, the pressure, temperature, and their interaction did not significantly affect the ζ-potential values of the beverages (*p*-value > 0.05), demonstrating that the SC–CO_2_ treatments did not modify the surface charge density, chemical structure of compounds, or molecular interactions of the functional araticum beverage. Recent studies have also shown the absence of an effect of SC–CO_2_ processing on the ζ-potential values of beverages, including an inulin-enriched soursop whey beverage [28] and inulin-enriched apple juice [21]. The ζ-potential values for the SC–CO_2_-treated beverages with the combination of different levels of pressure and temperature ranged from −32 to −36 mV. This was very close to the untreated beverage (−36 mV). The slight increase in ζ-potential values of the SC–CO_2_-treated beverages compared to the untreated beverage may be associated with conformational changes in the proteins present in the beverage due to mechanical (increase in pressure), thermal (increase in temperature), and/or acidic (reduction in pH) denaturation that occurred during the SC–CO_2_ processing [28]. Furthermore, our group has shown in previous studies that the chemical structure of fructooligosaccharides and inulin added to apple juice is not affected by SC–CO_2_ processing, maintaining the ζ-potential values before and after processing [21,22]. In fact, we subjected the functional araticum beverages to an analysis of inulin molecular profile and content by high-performance anion exchange chromatography coupled with pulsed amperometric detection (HPAEC–PAD). We did not find significant changes in these responses between the untreated and SC–CO_2_-treated beverages. These effects of the SC–CO_2_ processing on inulin structure will be discussed in more detail in Section 3.7.

The magnitude of the ζ-potential is indicative of the potential stability of a system because it results from the interaction between all charged molecules in the system, reflecting the degree of electrostatic repulsion or attraction between charged functional groups of the different molecules that make up the system [35]. Thus, the ζ-potential values can be used as an indicator of kinetic stability for beverages [36]. The functional araticum beverage showed negative ζ-potential values, indicating that the particles that make up the beverage are predominantly negatively charged. High absolute values of ζ-potential tend to keep liquid systems stable by preventing particle aggregation and flocculation. According to Pereira et al. [35], ζ-potential values with magnitudes greater than 30 mV, independent if positive or negative, produce stable colloidal systems during long-term storage. The ζ-potential values of untreated and SC–CO_2_-treated beverages ranged from −32 to −36 mV, suggesting good electrostatic stability of the system. Indeed, all beverages showed a good profile of physical stability during storage, as will be discussed in Section 3.5. The maintenance of this strong negative surface charge after all SC–CO_2_ treatments compared to the untreated beverage demonstrates that electrostatic repulsion can contribute to beverage stabilization.

### 3.4. Particle Size Distribution

**Figure 1:**
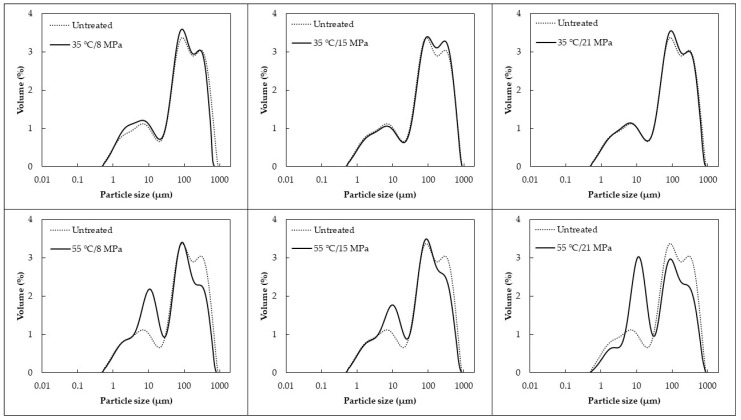
Effects of the SC–CO_2_ treatments on the particle size distribution of the functional araticum beverage.

The effects of SC–CO_2_ processing on the size distribution and particle size according to their Brouckere diameter (D_4,3_) of the functional araticum beverages are shown in Figure 1 and Table 1, respectively. The mean diameter (D_4,3_) of the particles suspended in the untreated beverage was 143 µm, while for SC–CO_2_-processed beverages, these values ranged between 105 and 148 µm. The size of particles suspended in the beverage was significantly modified by temperature (*p*-value < 0.001), pressure (*p*-value < 0.001), and the interaction between pressure and temperature (*p*-value = 0.002). The increase in temperature linearly reduced the size of the particles suspended in the beverage. On the other hand, the increase in pressure (from 8 to 15 MPa) initially promoted an increase in the size of particles suspended in the beverage, but the progressive increase in pressure (from 15 to 21 MPa) reduced the size of particles suspended in the beverage. Similar behavior to pressure was observed in the size of particles suspended in the beverage for the interaction between pressure and temperature, that is, regardless of the processing temperature used, an increase in particle size was observed when the pressure was raised from 8 to 15 MPa. In contrast, increasing the pressure from 15 to 21 MPa caused a reduction in these values.

The functional araticum beverage evaluated in this study is a complex and heterogeneous suspension composed of insoluble colloids and larger particles that are dispersed in an aqueous medium containing soluble compounds, including minerals, organic acids, sugars, and soluble particles (inulin). As the beverage was obtained from freeze-dried and crushed araticum pulp, the dispersed phase was mainly composed of cellular tissue fragments from the araticum pulp. The linear reduction in the size of particles suspended in the beverage as a result of increasing levels of SC–CO_2_ processing temperature (from 35 to 55 °C) may be associated with the cooking effect, which contributes to the increased solubilization of organic matter leading to a reduction in the diameter of suspended particles [21]. Meanwhile, at first, increasing the pressure level of the system (from 8 to 15 MPa), associated with the momentary acidification of the beverage during SC–CO_2_ processing, may promote the denaturation and aggregation of proteins, and the precipitation of proteins and charged polymers. This leads to the formation of aggregates and a consequent increase in the size of suspended particles [28]. However, the progressive increase in the pressure level (from 15 to 21 MPa) leads to an increase in the tension of the system during SC–CO_2_ processing. In turn, this causes the rupture of both protein/polymeric aggregates and cell wall fragments into smaller structures, promoting the reduction in the size of suspended particles in the beverage [21,37].

All functional araticum beverages showed a polydisperse particle distribution (Figure 1). Despite this similarity, the form and width of the curves differed from one beverage to another. All beverages, except for the beverage treated with SC–CO_2_ at 21 MPa and 55 °C, showed a high peak in the region of 100 µm. This was followed by a relatively high peak in the region of 1000 µm, and two smaller peaks in the regions of 1 and 10 µm. At the lowest temperature level (35 °C), there were hardly any noticeable differences in the particle size distribution profile between the untreated and SC–CO_2_-treated beverages as the system pressure was increased (from 8 to 21 MPa). However, increasing temperature (from 35 to 55 °C) and increasing pressure (from 8 to 21 MPa) associated with a higher processing temperature (55 °C) gradually reduced the two highest peaks, particularly the peak in the region of 1000 µm, while significantly increasing the peak in the region of 10 µm. Furthermore, these SC–CO_2_ processing conditions progressively shifted the curves towards smaller particle sizes. SC–CO_2_-treated beverage under the most intense processing conditions (21 MPa and 55 °C) presented two similar high peaks around 10 and 100 µm. One peak was relatively high in the region of 1000 µm and one peak was small in the region of 1 µm. As has been discussed previously, the intensification of SC–CO_2_ processing contributes to the increase in solubilization of organic matter and the rupture of cellular component fragments. This releases large amounts of small particles that lead to a reduction in the mean size of suspended particles in the processed beverage. The rupture of larger particles as SC–CO_2_ processing is intensified is evidenced in Figure 1 with a significantly lower particle size distribution near 1000 µm and a marked higher particle size distribution near 10 µm. This effect promotes a decrease in the polydispersity of the beverages, making them more homogeneous.

Functional araticum beverages presented polymodal size distribution (Figure 1), in which the characteristic peaks observed could be attributed to proteins (<1 µm), fat globules (1 to 10 µm), and araticum pulp fragmented material, insolubilized amorphous inulin crystals, and some coalesced fat globules (>10 µm). The particle distribution size of the dispersed phase can affect the physical stability of a beverage. According to Stokes’ Law, the velocity of movement of particles in a liquid system is directly proportional to the square of its radius. This means that the larger the size of the particles, the more unstable the liquid system can be [38]. As has been previously presented, the intensification of SC–CO_2_ processing gradually reduces the size of particles suspended in the beverage, particularly reducing the volume of particles near 1000 µm, while increasing the volume of particles in the region of 1 to 10 µm. This indicates the breakdown of larger particles into several smaller particles, suggesting higher stability and homogeneity of the beverages subjected to more intense SC–CO_2_ processing conditions. The effects of SC–CO_2_ treatments on the physical stability of the functional araticum beverage will be discussed in more depth in the following section (Section 3.5).

### 3.5. Kinetic Stability

Physical stability is another key parameter of fruit-based beverages. Thus, fruit-based beverages have been kinetically stabilized through processes that promote homogenization by reducing their particle size distribution. However, the addition of inulin may modify the stability characteristics of these products during storage [39]. Therefore, the Turbiscan assay was performed to examine the impact of the SC–CO_2_ treatments on the physical stability of the functional araticum beverage.

**Figure 2:**
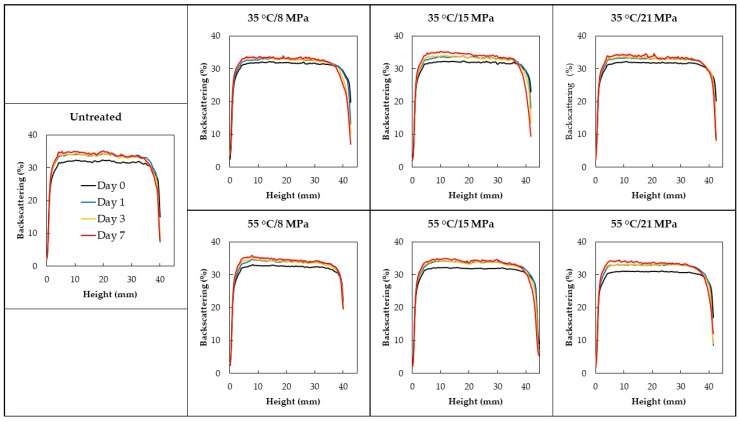
Effect of the SC–CO_2_ treatments on the backscattering profile of the functional araticum beverage during 7 days of cold storage at 5 ± 2 °C.

Figure 2 shows the impact of pressure and temperature, and their interaction on the physical stability of the functional araticum beverages right after their production (day 0) and after 1, 3, and 7 days of cold storage at 5 ± 2 °C. The results were obtained by performing a sweep of the bottom to the top of the glass tube containing the beverages and recording the backscattering profile (%) as a function of height (mm). All beverages, regardless of treatment and storage time, exhibited an almost constant backscattering profile along the height of the tube, indicating uniformity in the visual appearance of the beverage. The high physical stability of all beverages throughout the storage time, including the untreated beverage, was due to the gelling of the inulin, which was dissolved in hot water (80 °C) before being added to the beverage formulation. The addition of pre-gelatinized inulin promotes the gelling of the liquid system that composes the beverage, increasing the viscosity of the medium and creating a physical barrier against the coalescence of fat droplets and/or flocculation/sedimentation of particles from the araticum pulp [40].

During the storage time of the beverages, there was a slight and progressive increase in the backscattering values at the bottom and middle of the tube, with the same trend in the reduction in these values at the top of the tube, indicating the gradual destabilization of the beverage. These phenomena were most evident in the first 24 h of beverage storage (equilibrium time), after which, the backscattering values remained almost constant until day 7. The increase in backscattering values along almost the entire height of the tube over the storage time was related to the crystallization of inulin in the beverage. Meanwhile, the slight increase in backscattering values at the bottom of the tube (0–2 mm) over the storage time may have been due to the sedimentation of larger particles from the araticum pulp or inulin crystals [41]. As a result of these described phenomena, there was a slight clarification in the upper phase of the beverage over the storage time, reducing the backscattering values at the top of the tube (38–40 mm) due to a lower particle concentration in this region [37,42].

**Figure 3:**
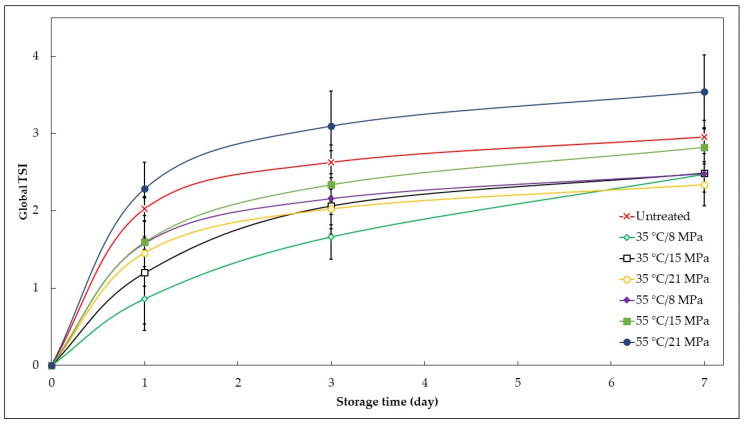
Effect of the SC–CO_2_ treatments on the phase separation kinetics of the functional araticum beverage during 7 days of cold storage at 5 ± 2 °C.

The impact of SC–CO_2_ treatments on the functional araticum beverage phase separation kinetic was quantitatively evaluated according to the global Turbiscan Stability Index (TSI) during 7 days of cold storage at 5 ± 2 °C. The results can be seen in Table 1 and Figure 3. The global TSI values result from the sum of all destabilization phenomena that occur in the beverage. Therefore, the higher the global TSI values, the greater the phase separation or destabilization of the beverage. The global TSI values of the SC–CO_2_-treated functional araticum beverages after 7 days of cold storage ranged from 2.3 to 3.5. These were significantly lower than those previously reported by Silva et al. [28] in an inulin-enriched soursop whey beverage processed with SC–CO_2_ and stored under the same conditions (7 days at 4 ± 2 °C), where the global TSI values ranged from 9 to 11.1. However, in the study by Silva et al. [28], a higher amount of inulin (6 g/100 g) was added along with a stabilizing agent (0.05 g of gellan gum/100 g). Nevertheless, the beverage developed here, with a lower amount of inulin (3 g/100 g) and the absence of a stabilizing agent, still showed significantly better kinetic stability after SC–CO_2_ processing. These results suggest that components of the araticum pulp may contribute to the stabilization of the beverage after SC–CO_2_ processing over storage time. Schiassi et al. [43] reported a considerable amount of pectin in the araticum pulp (1.22 g of total pectin/100 g fresh pulp). Pectin-containing solutions are widely recognized for their high gelling, thickening, and emulsifying capacities, improving the kinetic stability of food products [44]. Furthermore, the pectin present in the araticum pulp can interact with the added inulin in the beverage, leading to the formation of pectin/inulin-based structured systems that can improve the rheological characteristics of the beverage and, consequently, increase its kinetic stability. Indeed, Tarone et al. [45] found that the addition of inulin to pectin-based structured systems conferred greater apparent viscosity and consistency to these systems due to the ability of inulin to disrupt the microstructure of pectin and promote the formation of new and better-ordered pectin–inulin interactions, reducing the freedom of pectin polymer chains.

As can be observed in Figure 3, there was a progressive increase in global TSI values during storage time for all beverages, clearly indicating some degree of colloidal system destabilization over time. However, these destabilization processes mainly occurred within the first 24 h of storage, with a trend in stabilization in global TSI values after this period. Furthermore, all SC–CO_2_-processed beverages, except for the one treated under the most intense conditions (21 MPa and 55 °C), exhibited higher physical stability against phase separation during 7 days of cold storage compared to the untreated beverage, demonstrating the effectiveness of SC–CO_2_ technology in improving the physical stability of the beverage. The pressure and its interaction with the temperature did not influence the global TSI (*p*-value > 0.05) of the functional araticum beverage; however, the increase in temperature level increased the global TSI (*p*-value = 0.023). Therefore, the increase in temperature promoted a gradual phase separation in the beverages, likely as a result of particle aggregation. These outcomes may be related to the temperature’s ability, combined with the temporary pH reduction in the system, to promote fat globule coalescence; create high-molecular-weight complexes through the cross-linking of proteins, polysaccharides, or other components with the fat globule membrane; and induce protein coagulation due to thermal and/or acid denaturation [42,46,47]. Furthermore, the increase in temperature and acidity may have weakened the gel structure in the beverage (breaking/weakening inulin–inulin, inulin–pectin, and pectin–pectin interactions), facilitating the movement of suspended particles in the beverage and consequently leading to their coalescence/aggregation [48,49,50]. Thus, the weakening of the gel structure and the destabilization of fat globule membranes, and protein coagulation due to the synergistic effect of higher temperatures and a momentary lower pH during SC–CO_2_ processing promote progressive particle aggregation over the storage time, reducing the physical stability of the beverage. Despite the significant influence of temperature on global TSI on day 7 of cold storage, the instability difference indicated in the backscattering profiles (Figure 2) between SC–CO_2_-treated beverages was mild. There have been few studies evaluating the effect of SC–CO_2_ processing on the kinetic stability of fruit-based beverages, even less in fruit-based beverages enriched with inulin. Nonetheless, Silva et al. [28] reported similar results for the kinetic stability of an inulin-enriched soursop whey beverage after SC–CO_2_ processing.

### 3.6. Color Parameters

Color is another important indicator parameter for beverages because it influences the quality, commercial value, and acceptability of the final product [51]. Table 1 presents the effects of the SC–CO_2_ treatments on the instrumental color parameters, color index, yellow index, browning index, and color changes in the functional araticum beverage. According to the CIELAB pattern, the closer the colorimetric parameter values (L*, a*, and b*) are to zero, the more evident the neutral gray (achromatic) tonality becomes. Thus, the functional araticum beverage presented a clear appearance (L* values ranging from 53 to 55.6) and a yellowish color (b* values ranging from 20.2 to 21.1). Meanwhile, the values of the redness/greenness component (a*) had little influence on the color of the beverage, since they were close to zero for all samples (values ranging from 3.3 to 3.8). These color aspects were expected in the final product since the main components of the beverage have a light color: inulin is a white powder while araticum pulp is a yellowish powder. The yellowish color of the beverage is derived, at least in part, from the carotenoids present in the araticum pulp. Cardoso et al. [52] reported a high content of carotenoids in araticum pulp (4.98 mg/100 g fresh matter), with carotenes (α and β-carotene) being the predominant class, accounting for approximately 99.4% of the quantified carotenoids.

The pressure, temperature, and their interaction did not impact the color parameters of the beverage (*p*-value > 0.05). The absence of noticeable changes in the color of the beverage after SC–CO_2_ treatments was confirmed by evaluating the ΔE* values. The overall color difference (ΔE*) denotes a difference in color between each SC–CO_2_-treated beverage compared with the untreated beverage. According to Ramirez-Rodrigues et al. [53], consumers can only perceive a color change in a food product when the ΔE* value is greater than 3. However, all SC–CO_2_-treated beverages showed low ΔE* values (≤2), indicating that color changes provoked by SC–CO_2_ processing were imperceptible to the human eye.

Carotenoids are an important class of bioactive compounds present in the functional araticum beverage as, in addition to providing color, they are also responsible for a wide range of biological effects, including antioxidant capacity, pro-vitamin A activity, prebiotic-like effect, reduction in the risk of developing non-communicable chronic diseases, among others [5]. However, carotenoids can undergo isomerization and/or degradation reactions during food processing, negatively impacting their color and biological activity [54]. The maintenance of the b* values and yellow index after SC–CO_2_ processing indicates that the yellow color of the beverage was not modified, suggesting the absence of carotenoid degradation and/or isomerization.

The functional araticum beverage contains carbohydrates, proteins, and ascorbic acid. During beverage processing, particularly thermal processes, ascorbic acid can be degraded to furfural, while the carbonyl group of the carbohydrates can react with the amine group of the amino acids or proteins (Maillard reaction). In both cases, melanoidins are produced after successive reactions, culminating in the darkening of the beverage [55,56]. L* values and the browning index remained unchanged after all SC–CO_2_ treatments, demonstrating that the SC–CO_2_ processing conditions used do not promote non-enzymatic browning in the beverage.

These results suggest that SC–CO_2_ processing is an excellent alternative to thermal processing in beverages, as it is capable of maintaining color, and retaining important nutrients and bioactive compounds in the final product.

### 3.7. Sugars and Inulin Stability

The content and type of sugars are essential for the sensory, physicochemical, nutritional, and bioactive characteristics of plant-based beverages. An increase in the content of low-molecular-weight sugars (e.g., monosaccharides, disaccharides, and short-chain oligosaccharides) together with a reduction in the content of long-chain oligosaccharides and polysaccharides, may indicate that processing is hydrolyzing carbohydrates. On the other hand, a global reduction in carbohydrate content may suggest that processing is degrading carbohydrates through different reactions (e.g., oxidative and Maillard reactions). These reactions may cause unwanted changes in the sensory, physicochemical, nutritional, and bioactive properties (e.g., browning, reduction in nutritional and prebiotic properties, destabilization of the colloidal system, and imbalance between sweetness and sourness, among others) of processed food [18,39]. Therefore, evaluating the effect of SC–CO_2_ processing on the profile and content of sugars in functional araticum beverages can provide important information regarding the best process conditions to preserve its sensory, physicochemical, nutritional, and bioactive characteristics, ensuring food safety.

The contents of monosaccharides and disaccharides of the functional araticum beverage before and after SC–CO_2_ treatments were determined by HPAEC–PAD. As is shown in Table 2, only glucose and fructose were identified in the beverage. As was previously reported by Arruda et al. [57], glucose and fructose are the main sugars present in araticum pulp and their ratio is nearly 1:1 (1.00:1.06 of glucose:fructose). However, in the functional araticum beverage developed here, this ratio was significantly modified to 1.00:1.19 of glucose:fructose, indicating the presence of a relatively high amount of fructose in the inulin used to enrich the beverage. The variables of SC–CO_2_ processing (pressure, temperature, and their interaction) did not affect the content of glucose and fructose present in the beverage (*p*-value > 0.05). Cappelletti et al. [58], Silva et al. [21], and Silva et al. [22] also reported no effect of SC–CO_2_ processing on the contents of glucose and fructose in coconut water (12 MPa at 40 °C for 30 min), inulin-enriched apple juice (10–20 MPa and 67% CO_2_ volume ratio at 35 °C for 10 min), and FOS-enriched apple juice (8–21 MPa and 20–50% CO_2_ volume ratio at 40–60 °C for 10 min), respectively.

**Figure 4:**
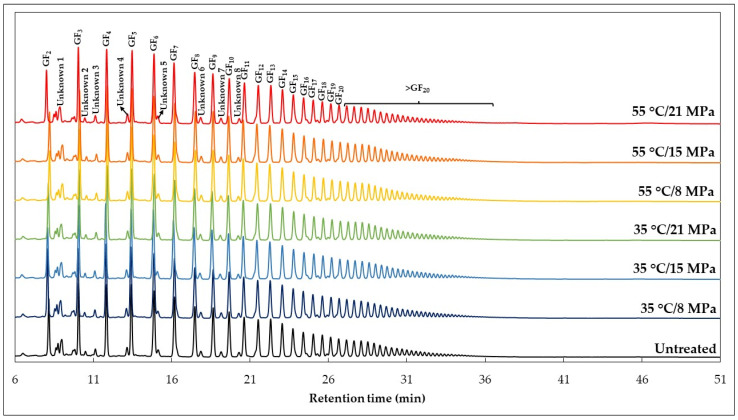
Effect of the SC–CO_2_ treatments on the inulin chromatographic profile of the functional araticum beverage.

Figure 4 shows the chromatographic profile of oligosaccharides obtained by HPAEC–PAD in the functional araticum beverage before and after SC–CO_2_ treatments. Fructan-type chains with different degrees of polymerization, certainly derived from inulin, were identified in the beverage, as well as some peaks related to unknown compounds. We believe that these unknown peaks are also compounds derived from inulin, since araticum pulp does not contain substantial amounts of oligosaccharides, as described by Arruda et al. [57]. Inulin is a linear fructan-type polysaccharide that is composed of successive fructose units linked together by β-(2→1) bonds (F*n*), usually with a terminal glucose unit linked to the chain by an α-(1↔2) bond (GF*n*). Thus, inulin can be predominantly of the F*n* or GF*n* type, with a degree of polymerization ranging from 2 to 60 depending on the plant source [18]. The inulin used to enrich the functional araticum beverage was extracted from chicory root. Chicory native inulin is known to be composed mainly of a mixture of GF*n*-type fructan chains [59]. Therefore, we suppose that these low-intensity unknown peaks are referring to F*n*-type fructan chains that are produced in small amounts due to the slight hydrolysis of native inulin during the extraction and purification processes, as they were already present in the beverage before undergoing SC–CO_2_ processing.

As can be observed in Figure 4, the inulin present in the untreated beverage showed a chromatographic profile very similar to those treated with SC–CO_2_. No additional peaks were identified after the SC–CO_2_ treatments, demonstrating that no distinct sugars were formed, apart from those already present in the matrices that made up the beverage. Moreover, there was no suppression or intensification of the peaks identified in the beverage after SC–CO_2_ treatments, suggesting that the added inulin was not degraded by the SC–CO_2_ processing. The same behavior was observed for inulin-enriched apple juice and fructooligosaccharide-enriched apple juice treated with non-thermal SC–CO_2_ technology [21,22].

Although the qualitative chromatographic profile of inulin provided sufficient evidence to prove the absence of inulin degradation in the SC–CO_2_-treated beverages, quantitative analyses of the fructooligosaccharides that make up inulin were performed and the results are presented in Table 2 and Table 3. Only a few short-chain fructooligosaccharides (GF_2_–GF_4_) were quantified based on analytical curves due to the absence of commercial standards for higher degrees of polymerization. Thus, the other GF*n*-type fructans (≥ GF_5_) and unknown compounds (possibly F*n*-type fructans) were quantitatively analyzed based on the peak area, maintaining the same injection conditions into the HPAEC–PAD system (All samples were diluted 100-fold for injection into the chromatographic system). None of the SC–CO_2_ process variables (pressure, temperature, and their interaction) affected the content of GF*n*-type fructans present in the beverage (*p*-value > 0.05). However, a slight significant increase was observed in the contents of three unknown oligosaccharides (unknown compounds 6 (*p*-value = 0.03), 7 (*p*-value = 0.029), and 8 (*p*-value = 0.03)) as the SC–CO_2_ processing temperature was increased from 35 to 55 °C. As was discussed previously, these unknown oligosaccharides, detected in low quantities in the beverage, can be F*n*-type fructans. These are possibly generated due to the partial hydrolysis of native inulin during the extraction and purification steps. Several studies have demonstrated that during the thermal treatment of fruit-based beverages, partial hydrolysis of inulin may occur, mainly due to the low pH of these liquid systems. This, when associated with high temperatures, favors the depolymerization of inulin, leading to an increase in the quantities of low-molecular-weight GF*n*-type and F*n*-type fructans and, consequently, a reduction in those that are high molecular weight [21,22,60,61]. Although changes in the content of most of the fructan chains that make up inulin were not observed in the present study, the small increase in the content of the unknown oligosaccharides (compounds 6, 7, and 8) may be due to the slight hydrolysis of inulin. This is caused by the synergistic effect between thermal and chemical stress due to the temporary reduction in the beverage’s pH during SC–CO_2_ processing. Likewise, Silva et al. [22] observed a slight increase in the content of some short-chain fructooligosaccharides (GF_3_ and GF_4_) in fructooligosaccharide-enriched apple juice after SC–CO_2_ processing at 60 °C (8–21 MPa, 20–50% CO_2_ volume ratio, and 10 min). However, they did not notice any modifications in fructooligosaccharides content when it was processed at a lower temperature (40 °C). Silva et al. [21] also did not report any changes in the profile and content of fructan chains in inulin-enriched apple juice treated with non-thermal SC–CO_2_ (35 °C, 10–20 MPa, and 10 min). These results reinforce the synergistic effect between thermal and acid stress on inulin depolymerization in SC–CO_2_-treated beverages, since at low processing temperatures, only the temporary reduction in the system’s pH is not sufficient to cause inulin degradation. Therefore, our results, together with those reported in the literature, demonstrate that non-thermal SC–CO_2_ processing does not affect the sugar and inulin stability in beverages, evidencing the viability of this emerging technology in the industrial stabilization of food products.

## 4. Conclusions

The stabilization of the functional araticum beverage by using non-thermal SC–CO_2_ technology and the establishment of the effects of SC–CO_2_ processing variables (pressure and temperature) on the sensory, physicochemical, nutritional, and functional properties of the beverage were successfully conducted. Furthermore, the addition of inulin to the araticum beverage provided an innovative functional fruit-based beverage due to its high content of prebiotic carbohydrates. The use of SC–CO_2_ technology as a stabilizing technique for the functional araticum beverage has proven to be viable from a sensory, nutritional, and functional standpoint, as the physicochemical properties (total soluble solids, pH, and ζ-potential), color parameters, and the content and profile of functional compounds (particularly inulin) in the beverage were minimally affected or even unchanged by the treatment conditions employed in the present study. On the other hand, SC–CO_2_ technology altered the particle size distribution of the beverage, as process intensification led to the formation of particles with a smaller mean diameter such as those observed in other high-pressure-based stabilization techniques. It is also important to highlight that SC–CO_2_ processing preserved the profile and content of fructan-type oligosaccharides that make up the added inulin in the beverage. Therefore, the processing of the functional araticum beverage using SC–CO_2_ technology resulted in products with very similar characteristics to the untreated product. Moreover, SC–CO_2_ processing proved to be effective in improving the physical stabilization of the beverage, minimizing phase separation during cold storage. This can enable its availability in the market without the formation of bottom body and/or clarified areas, which are undesirable characteristics for consumers. Therefore, the results obtained here have contributed to the consolidation of SC–CO_2_ technology as a viable alternative, from a technological standpoint, for processing fruit-based beverages enriched with prebiotic carbohydrates. This establishes its potential for value addition of these products by preserving their sensory, physicochemical, nutritional, and functional characteristics, while improving their physical stability during storage. However, sensory analyses should be conducted in future studies to determine the optimal formulation of the beverage as well as the actual impact of SC–CO_2_ processing on the sensory aspects.

## Figures and Tables

**Table 1:** Effect of SC–CO_2_ treatments on the physicochemical properties, kinetic stability, and color parameters of the functional araticum beverage.

Parameter		SC–CO_2_ Treatments					
		35 °C			55 °C		
	Untreated	8 MPa	15 MPa	21 MPa	8 MPa	15 MPa	21 MPa
pH	4.5 ± 0.1	4.43 ± 0.01	4.46 ± 0.01	4.52 ± 0.01	4.54 ± 0.02	4.51 ± 0.01	4.5 ± 0.1
TSS (°Brix)	7.4 ± 0.1	7.4 ± 0.1	7.1 ± 0.1	7.3 ± 0.2	7.4 ± 0.1	7.5 ± 0.2	7.4 ± 0.1
ζ-potential (mV)	−36 ± 1	−35.1 ± 0.4	−32 ± 3	−35 ± 2	−36 ± 2	−34 ± 1	−35 ± 1
D_4,3_ (µm)	143 ± 6	121 ± 7	148 ± 6	136 ± 4	105 ± 5	114 ± 2	106 ± 3
Global TSI (day 7)	3.0 ± 0.1	2.5 ± 0.2	2.5 ± 0.3	2.3 ± 0.3	2.48 ± 0.03	2.8 ± 0.4	3.5 ± 0.5
L*	55.6 ± 0.3	55 ± 1	54.9 ± 0.4	55.2 ± 0.3	54.6 ± 0.5	54.8 ± 0.4	53 ± 1
C*	20.6 ± 0.3	21 ± 1	21.4 ± 0.2	21.2 ± 0.1	20.66 ± 0.03	21.3 ± 0.1	20.5 ± 0.4
h	81 ± 1	81 ± 1	80.2 ± 0.1	80.7 ± 0.1	80.2 ± 0.4	79.7 ± 0.2	80.0 ± 0.3
a*	3.3 ± 0.3	3.4 ± 0.6	3.64 ± 0.01	3.4 ± 0.1	3.5 ± 0.1	3.8 ± 0.1	3.53 ± 0.02
b*	20.5 ± 0.1	21 ± 1	21.1 ± 0.2	20.97 ± 0.02	20.4 ± 0.1	21.0 ± 0.1	20.2 ± 0.4
Color index	2.8 ± 0.1	2.9 ± 0.2	2.98 ± 0.02	2.92 ± 0.04	2.9 ± 0.1	3.0 ± 0.1	3.0 ± 0.1
Yellow index	53 ± 1	54 ± 3	54.9 ± 0.2	54.3 ± 0.3	53.3 ± 0.3	55 ± 1	54.0 ± 0.1
Browning index	49 ± 1	50 ± 4	51.7 ± 0.2	50.6 ± 0.4	50 ± 1	52 ± 1	50.64 ± 0.03
ΔE*	-	1.7 ± 0.3	1.4 ± 0.1	0.7 ± 0.3	1.3 ± 0.6	1.6 ± 0.4	2 ± 1

TSS: total soluble solids; D_4,3_: mean particle size; TSI: Turbiscan Stability Index; L*: represents the lightness with values from 0 (black) to 100 (white); C*: represents the chromaticity; h: represents the hue of the color with values from 0° (red) to 270° (blue); a*: represents redness/greenness where positive values are red and negative values are green; b*: represents yellowness/blueness where positive values are yellow and negative values are blue; ΔE*: color difference between the SC–CO_2_-processed and untreated beverages.

**Table 2:** Effect of SC–CO_2_ treatments on the content of sugars and short-chain fructooligosaccharides (mg/mL) of the functional araticum beverage.

Sugar	r.t. (min)		SC–CO_2_ Treatments					
			35 °C			55 °C		
		Untreated	8 MPa	15 MPa	21 MPa	8 MPa	15 MPa	21 MPa
Glucose	4.42	13.6 ± 0.4	14.1 ± 0.5	14.2 ± 0.2	14.0 ± 0.1	13.8 ± 0.3	14.4 ± 0.1	14.1 ± 0.3
Fructose	4.96	16.2 ± 0.5	17 ± 1	16.9 ± 0.1	16.4 ± 0.3	16.4 ± 0.4	17.0 ± 0.1	17.0 ± 0.3
GF_2_	8.14	0.67 ± 0.02	0.68 ± 0.03	0.64 ± 0.01	0.65 ± 0.03	0.6 ± 0.1	0.64 ± 0.04	0.65 ± 0.04
GF_3_	10.07	0.79 ± 0.02	0.78 ± 0.01	0.78 ± <0.01	0.77 ± 0.01	0.78 ± <0.01	0.77 ± 0.02	0.79 ± 0.01
GF_4_	11.84	1.05 ± <0.01	1.05 ± 0.01	1.06 ± <0.01	1.04 ± 0.01	1.06 ± <0.01	1.05 ± 0.03	1.06 ± 0.02

r.t.: retention time; GF_2_: 1-kestose; GF_3_: 1-nystose; GF_4_: 1-fructofuranosylnystose.

**Table 3:** Effect of SC–CO_2_ treatments on the molecular profile of fructooligosaccharides from the inulin added to the functional araticum beverage regarding their peak area (nC*min).

FOS	r.t. (min)		SC–CO_2_ Treatments					
			35 °C			55 °C		
		Untreated	8 MPa	15 MPa	21 MPa	8 MPa	15 MPa	21 MPa
GF_2_	8.14	15.9 ± 0.4	16 ± 1	15.2 ± 0.1	15 ± 1	15.2 ± 0.5	15.0 ± 0.1	15 ± 1
Uk 1	8.96	5.34 ± 0.03	5.4 ± 0.1	5.5 ± 0.1	5.4 ± 0.1	5.5 ± 0.2	5.5 ± 0.1	5.5 ± 0.2
GF_3_	10.07	18.6 ± 0.4	18.2 ± 0.1	18.4 ± 0.1	18.1 ± 0.1	18.3 ± 0.1	18.1 ± 0.4	18.5 ± 0.4
Uk 2	10.49	1.1 ± 0.1	1.10 ± 0.02	1.10 ± 0.03	1.09 ± 0.03	1.12 ± 0.02	1.08 ± 0.03	1.1 ± 0.1
Uk 3	11.14	2.5 ± 0.1	2.5 ± 0.1	2.5 ± 0.1	2.5 ± 0.1	2.5 ± 0.1	2.5 ± 0.1	2.6 ± 0.1
GF_4_	11.84	21.6 ± 0.1	21.6 ± 0.2	21.9± 0.1	21.5 ± 0.2	21.8 ± 0.1	22 ± 1	21.9 ± 0.4
Uk 4	13.14	2.37 ± 0.02	2.39 ± 0.02	2.41 ± 0.02	2.38 ± 0.03	2.45 ± 0.02	2.4 ± 0.1	2.5 ± 0.1
GF_5_	13.42	20.8 ± 0.1	20.8 ± 0.1	21.1 ± 0.1	20.7 ± 0.1	21.0 ± 0.1	20.9 ± 0.4	21.1 ± 0.3
GF_6_	14.86	21.9 ± 0.4	21.9 ± 0.3	22.3 ± 0.1	21.8 ± 0.2	22.2 ± 0.3	22.0 ± 0.5	22.3 ± 0.3
Uk 5	15.12	1.4 ± 0.2	1.4 ± 0.2	1.6 ± 0.1	1.4 ± 0.1	1.5 ± 0.2	1.6 ± 0.1	1.5 ± 0.1
GF_7_	16.17	22.1 ± 0.2	21.9 ± 0.2	22.3 ± 0.1	21.9 ± 0.3	22.3 ± 0.1	22.2 ± 0.4	22.5 ± 0.4
GF_8_	17.49	20.0 ± 0.1	19.9 ± 0.2	19.1 ± 0.1	19.8 ± 0.1	19.1 ± 0.1	20.0 ± 0.4	19.2 ± 0.3
Uk 6	17.87	2.05 ± 0.02	2.05 ± 0.02	2.09 ± 0.01	2.05 ± 0.01	2.15 ± 0.04	2.1 ± 0.1	2.2 ± 0.1
GF_9_	18.64	18.2 ± 0.2	18.1 ± 0.2	18.4 ± 0.1	18.1 ± 0.2	18.3 ± 0.1	18.2 ± 0.4	18.3 ± 0.2
Uk 7	19.16	2.01 ± 0.02	2.01 ± 0.03	2.05 ± 0.01	2.01 ± <0.01	2.11 ± 0.03	2.07 ± 0.04	2.1 ± 0.1
GF_10_	19.68	16.9 ± 0.2	16.9 ± 0.2	17.1 ± 0.1	16.8 ± 0.2	17.1 ± 0.1	17.0 ± 0.4	17.1 ± 0.1
Uk 8	20.31	1.42 ± 0.03	1.39 ± 0.02	1.44 ± 0.01	1.40 ± 0.04	1.46 ± 0.02	1.46 ± 0.04	1.5 ± 0.1
GF_11_	20.63	15.0 ± 0.3	14.9 ± 0.2	15.2 ± 0.3	14.9 ± 0.4	15.2 ± 0.1	15.2 ± 0.4	15.1 ± 0.1
GF_12_	21.50	15.9 ± 0.3	15.9 ± 0.2	16.1 ± 0.2	15.9 ± 0.2	16.2 ± 0.2	16.1 ± 0.4	16.1 ± 0.1
GF_13_	22.31	14.6 ± 0.3	14.7 ± 0.2	14.8 ± 0.3	14.7 ± 0.2	15.0 ± 0.1	14.9 ± 0.4	14.8 ± 0.2
GF_14_	23.08	13.1 ± 0.3	13.2 ± 0.2	13.3 ± 0.3	13.2 ± 0.2	13.5 ± 0.2	13.4 ± 0.4	13.3 ± 0.1
GF_15_	23.78	12.1 ± 0.3	12.2 ± 0.2	12.3 ± 0.3	12.3 ± 0.2	12.5 ± 0.2	12.4 ± 0.3	12.2 ± 0.1
GF_16_	24.44	10.4 ± 0.4	10.6 ± 0.1	10.5 ± 0.3	10 ± 1	10.8 ± 0.1	10.8 ± 0.3	10.6 ± 0.1
GF_17_	25.06	8.6 ± 0.3	8.4 ± 0.1	8.4 ± 0.3	8.5 ± 0.1	8.5 ± 0.1	8.5 ± 0.3	8.3 ± 0.2
GF_18_	25.64	7.7 ± 0.3	7.9 ± 0.1	7.9 ± 0.3	7.9 ± 0.1	8.0 ± 0.2	8.0 ± 0.2	7.7 ± 0.2
GF_19_	26.19	6.8 ± 0.3	7.0 ± 0.1	7.0 ± 0.3	7.0 ± 0.1	7.1 ± 0.2	7.1 ± 0.2	6.8 ± 0.2
GF_20_	26.71	7.5 ± 0.3	7.7 ± 0.1	7.7 ± 0.3	7.7 ± 0.1	7.8 ± 0.2	7.7 ± 0.2	7.6 ± 0.2
GF_21_	27.20	7.2 ± 0.4	7.3 ± 0.1	7.3 ± 0.3	7.4 ± 0.1	7.5 ± 0.2	7.5 ± 0.2	7.2 ± 0.2
GF_22_	27.67	6.5 ± 0.3	6.7 ± 0.1	6.7 ± 0.3	6.8 ± 0.1	6.8 ± 0.2	6.9 ± 0.2	6.6 ± 0.2
GF_23_	28.11	6.1 ± 0.3	6.3 ± 0.1	6.3 ± 0.3	6.3 ± 0.1	6.4 ± 0.2	6.4 ± 0.2	6.2 ± 0.2
GF_24_	28.54	5.3 ± 0.3	5.5 ± 0.1	5.5 ± 0.3	5.5 ± 0.1	5.6 ± 0.2	5.6 ± 0.2	5.4 ± 0.2
GF_25_	28.95	4.9 ± 0.3	5.1 ± 0.1	5.1 ± 0.3	5.2 ± 0.1	5.2 ± 0.1	5.2 ± 0.2	4.9 ± 0.2
GF_26_	29.33	4.5 ± 0.3	4.7 ± 0.1	4.7 ± 0.3	4.7 ± 0.1	4.8 ± 0.2	4.8 ± 0.2	4.6 ± 0.2
GF_27_	29.70	3.9 ± 0.3	4.1 ± 0.1	4.0 ± 0.2	4.1 ± 0.1	4.1 ± 0.1	4.2 ± 0.2	3.9 ± 0.2
GF_28_	30.05	3.3 ± 0.2	3.4 ± 0.1	3.4 ± 0.2	3.4 ± 0.1	3.4 ± 0.1	3.5 ± 0.1	3.3 ± 0.2
GF_29_	30.39	2.6 ± 0.2	2.8 ± 0.1	2.7 ± 0.2	2.8 ± 0.1	2.8 ± 0.1	2.8 ± 0.1	2.6 ± 0.2
GF_30_	30.72	2.1 ± 0.2	2.27 ± 0.03	2.2 ± 0.2	2.30 ± 0.04	2.3 ± 0.1	2.3 ± 0.1	2.1 ± 0.2
GF_31_	31.03	1.8 ± 0.2	1.94 ± 0.03	1.9 ± 0.2	1.95 ± 0.02	1.9 ± 0.1	2.0 ± 0.1	1.8 ± 0.2
GF_32_	31.34	1.7 ± 0.2	1.77 ± 0.02	1.7 ± 0.2	1.79 ± 0.01	1.8 ± 0.1	1.8 ± 0.1	1.6 ± 0.2
GF_33_	31.63	1.5 ± 0.2	1.65 ± 0.03	1.6 ± 0.2	1.67 ± 0.01	1.7 ± 0.1	1.7 ± 0.1	1.5 ± 0.2
GF_34_	31.91	1.4 ± 0.2	1.53 ± 0.01	1.5 ± 0.2	1.55 ± 0.01	1.5 ± 0.1	1.6 ± 0.1	1.4 ± 0.1
GF_35_	32.19	1.3 ± 0.2	1.40 ± 0.02	1.4 ± 0.1	1.42 ± 0.01	1.4 ± 0.1	1.5 ± 0.1	1.3 ± 0.1
GF_36_	32.45	1.2 ± 0.2	1.27 ± 0.02	1.2 ± 0.1	1.29 ± 0.01	1.3 ± 0.1	1.3 ± 0.1	1.2 ± 0.1
GF_37_	32.71	1.0 ± 0.2	1.14 ± 0.01	1.1 ± 0.1	1.15 ± 0.01	1.2 ± 0.1	1.2 ± 0.1	1.0 ± 0.1
GF_38_	32.96	0.9 ± 0.2	1.00 ± 0.01	1.0 ± 0.1	1.02 ± <0.01	1.0 ± 0.1	1.05 ± 0.04	0.9 ± 0.1
GF_39_	33.20	0.8 ± 0.1	0.87 ± 0.01	0.9 ± 0.1	0.89 ± <0.01	0.9 ± 0.1	0.91 ± 0.04	0.8 ± 0.1
GF_40_	33.44	0.7 ± 0.1	0.75 ± 0.01	0.7 ± 0.1	0.77 ± <0.01	0.8 ± 0.1	0.79 ± 0.03	0.7 ± 0.1

r.t.: retention time; FOS: fructooligosaccharide; Uk: unknown compound.

## Data Availability

Data are contained within the article.
